# The C825T Polymorphism of the G-Protein *β*3 Gene as a Risk Factor for Functional Dyspepsia: A Meta-Analysis

**DOI:** 10.1155/2016/5037254

**Published:** 2015-12-31

**Authors:** Yi-Zuo Song, He-Yi You, Zhe-Hui Zhu, Zheng-De Wen, Hui-Ying Xu, Bi-Cheng Chen, Zong-Jing Chen, Qing-Ke Huang

**Affiliations:** ^1^Zhejiang Provincial Top Key Discipline in Surgery, Wenzhou Key Laboratory of Surgery, Department of Surgery, The First Affiliated Hospital, Wenzhou Medical University, Wenzhou, Zhejiang 325000, China; ^2^Department of Gastroenterology, The First Affiliated Hospital, Wenzhou Medical University, Wenzhou, Zhejiang 325000, China

## Abstract

*Background*. Functional dyspepsia (FD) is a functional upper gastrointestinal disorder with significant morbidity and medical costs. Previous studies investigated the association of G-protein *β*3 (GNB3) genetic polymorphisms with FD but with inconsistent results. Therefore, we performed a meta-analysis to derive a precise estimation of the relationship between GNB3 polymorphisms and FD.* Methods*. We searched different databases including PubMed, EMBASE, CNKI, and the Ovid Library to gather eligible studies on GNB3 polymorphisms and FD. The association was assessed by the odds ratio (OR) with 95% confidence intervals (CI).* Results*. We identified 12 studies with 1109 cases and 2853 controls for the analysis. We found no associations of GNB3 C825T polymorphism with FD in the overall population (T versus C, OR = 1.06, 95% CI: 0.96–1.18, *P* = 0.26; TT versus CC + CT, OR = 1.16, 95% CI: 0.97–1.39, *P* = 0.11; TT + CT versus CC, OR = 1.01, 95% CI: 0.77–1.31, *P* = 0.96; TT versus CC, OR = 1.15, 95% CI: 0.93–1.44, *P* = 0.20). Subgroup analyses by genotyping method indicated that the magnitude of association was strengthened for additive model (OR = 1.15, 95% CI: 1.07–2.24, *P* = 0.02). Sensitivity analysis did not reveal significant associations under all models.* Conclusions*. This meta-analysis demonstrates that GNB3 C825T polymorphism may not be a risk factor for FD.

## 1. Introduction

Functional dyspepsia is one of the most common chronic gastrointestinal disorders encountered in clinical practice [1], with prevalence up to 40% and annual incidence ranging from 1% to 6% in population-based studies, respectively [2–5]. Both in uninvestigated dyspepsia in the general population and in patients with functional dyspepsia who are seen in tertiary-care settings, the most typical dyspeptic symptoms include postprandial fullness, upper abdominal bloating, epigastric pain, and early satiation, often accompanied by other upper gastrointestinal symptoms such as nausea, belching, or epigastric burning. In accordance with the Rome III consensus, the subdivision of FD was proposed into postprandial distress syndrome (PDS) characterized by postprandial fullness and early satiation, and epigastric pain syndrome (EPS) with characteristics of epigastric pain or burning [6]. Health-related quality of life has been considerably reduced in patients with dyspepsia due to their symptoms, particularly abdominal pain and indigestion, causing emotional distress, problems with food and drink, and impaired vitality. Meanwhile, FD constitutes a major effect on heavy economic burdens by demanding extensive medical care and diagnostic procedures [7, 8].

However, the pathogenesis contributing to the huge amount of intermittent functional dyspepsia currently remains unclear. FD is a multifactorial disease which is likely to be the consequence of genetic factors, environmental stimuli, and their interaction. Several possible etiological factors, including abnormal gastrointestinal motility, visceral hypersensitivity, delayed gastric emptying, dysfunction of the autonomic nervous system,* Helicobacter pylori* (*H. pylori*) infection, and serum ghrelin level, are repeatedly announced to be implicated in the pathogenesis of FD [9–14]. In addition, psychological disturbances together with lifestyle factors have been proven to be associated with the development of this complex disease such as smoking, anxiety, and depression [15–17]. Nevertheless, the phenomenon that FD cases cluster in families has demonstrated a strong heritability for FD, suggesting that genetic factors may play a more crucial role than environmental ones in the pathophysiologic mechanisms of FD [18, 19].

G-proteins are heterotrimers composed of *α*, *β*, and *γ* subunits, which act as switches for signal transduction from extracellular space into the cell and are expressed in all cells of the human body [20]. Concretely, the heterotrimeric G-protein, via interacting with G-protein coupled receptors, can be dissociated into G*α*-GTP and G*βγ* complexes, resulting in a variety of complex physiological responses such as cell proliferation, chemotaxis, and vasoconstriction [21]. G-protein *β*3 (GNB3) subunit plays a vital role on several signal transduction receptors and effectors, encoded by the GNB3 gene located on chromosome 12p13 which comprises 11 exons and 10 introns [21, 22]. Reportedly, one widely studied polymorphism of the GNB3 gene is the C825T (rs5443) polymorphism, which consists of a substitution of C by T as position 825 in exon 10 [22]. Additionally, the C825T polymorphism was found to be associated with a shortened splice variant of the GNB3 protein that gives rise to enhanced signal transduction via pertussis toxin-sensitive G-proteins, while the 825T allele appears to cause alternative splicing of the gene, thereby contributing to the generation of a functionally active splice variant, referred to as GNB3s [23].

To date, association between GNB3 C825T polymorphism and FD has been extensively pursued with contradictory results in several epidemiological studies [24–27]. Generally, different population structures, inadequate sample size, and ethnic differences might account for the observed inconsistency. Therefore, we performed a meta-analysis of all available data to systematically and effectively clarify the association between the GNB3 C825T polymorphism and FD risk.

## 2. Materials and Methods

### 2.1. Search Strategy

To search for all the studies investigating the association of the GNB3 polymorphism with FD risk, we conducted a systematic computerized literature search from PubMed, EMBASE, the Ovid Library, and the Chinese National Knowledge Infrastructure (CNKI) prior to March 2015 using the following keywords and subject terms: “functional dyspepsia” or “FD,” “G-protein beta3” or “GNB3,” “mutation,” “variant,” and “polymorphism.” The full text of the retrieved articles was scrutinized to inspect whether information on the topic of interest was included. To ensure a comprehensive acquisition of literature, independent supplemental manual searches were established on the reference lists of the retrieved articles, finding additional studies that were not identified initially. To avoid the local literature bias, the search was diffusely designed without language and region restrictions. The literature retrieval was performed independently by two investigators and discrepancies were resolved by reaching a consensus or input from a third investigator if required.

### 2.2. Inclusion and Exclusion Criteria

As a prerequisite, the selection of studies in our meta-analysis was abided by the predefined inclusion and exclusion criteria: (1) population-based or hospital-based case-control or cohort studies evaluating the relationship between the GNB3 C825T polymorphism and functional dyspepsia; (2) sufficient data on genotypic and allelic frequencies between FD patients and controls for estimating an odds ratio (OR) with a 95% confidence interval (CI); and (3) studies with related clinical characteristics limited to those using human subjects and containing demographic information of subjects. To avoid selection bias, articles regarding controls in which the genotype distribution deviated from the Hardy-Weinberg equilibrium (HWE) and cases simultaneously compounded with another disease, such as irritable bowel syndrome (IBS), were also included. Two reviewers then assessed the full text of the retrieved studies independently for their suitability of inclusion. Any discrepancies concerning articles meriting inclusion between reviewers were resolved by a consensus meeting of three authors.

### 2.3. Data Extraction

To make sure of the accuracy of the data, the data were independently gathered in duplicate by two investigators on the basis of a standard protocol. From each qualified study the following data were abstracted: the first author, the year of publication, FD diagnostic criteria, genotyping method, baseline characteristics of the study population (country, ethnicity, mean age, and sex), total numbers of cases and controls and the genotype distribution, and the Hardy-Weinberg equilibrium (HWE) using the *χ* ^2^ test. Any encountered discrepancies were adjudicated by a discussion until a consensus was reached.

### 2.4. Quality Score Assessment

The quality of each selected study was assessed independently by the same two investigators according to the Newcastle-Ottawa Scale (NOS) (http://www.ohri.ca/programs/clinical_epidemiology/oxford.asp). Scores were based on the selection, comparability, and exposure (case-control studies) or outcome (cohort studies) of the studies. To avoid selection bias, studies of poor quality were not rejected in this meta-analysis.

### 2.5. Statistical Analysis

Before estimating the relationship between the GNB3 C825T polymorphism and FD risk, a test that detects whether the genotype frequencies of the controls were in HWE was applied to evaluate the data quality, using a *χ* ^2^ test (*P* > 0.05) [28]. Odds ratios (ORs) with 95% confidence interval (CI) were calculated to assess the intensity of the association between the GNB3 C825T polymorphism and FD. The heterogeneity among studies was appraised by the Cochran *Q* test. In addition to a visual assessment using the forest plots, we calculated the inconsistency index (*I* ^2^) to quantify the extent of between-study variability, which represented the proportion of heterogeneity not explained by random variation [29]. Statistically significant heterogeneity was considered present at *I* ^2^ > 50% (*I* ^2^ = 0–25%, no heterogeneity; *I* ^2^ = 25–50%, moderate heterogeneity; *I* ^2^ = 50–75%, large heterogeneity; *I* ^2^ = 75–100%, extreme heterogeneity) [29]. When the *Q* test was significant (*P* < 0.05) or *I* ^2^ > 50%, indicating the presence of heterogeneity, a random-effects model (the DerSimonian and Laird method) was used [30]; otherwise, the fixed-effects model (the Mantel-Haenszel method) was used [31]. To explore the latent source of heterogeneity, we conducted a subgroup analysis by preestablishing several potential covariates including the definition of FD, region, and genotyping method. Furthermore, in an attempt to look at more narrowly drawn subsets of the studies whose quality score was lower than 6 or controls deviated from the HWE or cases represented concomitant symptoms of FD and IBS, separate analyses were undertaken in a sensitive way. Finally, we constructed funnel plots and performed Egger's test for publication bias by inspecting the symmetry of funnel plots, with *P* < 0.05 for Egger's test indicating significant publication bias [28]. All tests were two-sided and a *P* value < 0.05 was defined as statistical significance. All statistical analyses were conducted using Stata 12.0 (Stats Corp, College Station, TX, USA) and Review Manager 5.0 (The Cochrane Collaboration, Oxford, UK).

## 3. Results

### 3.1. Literature Search and Study Characteristics

**Figure 1:**
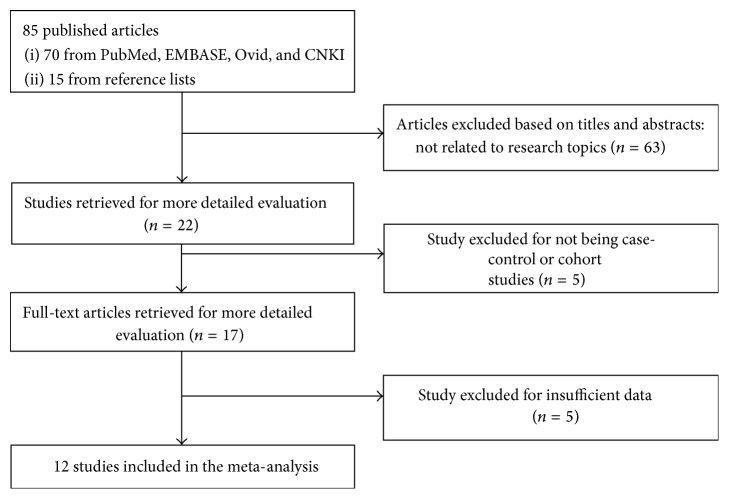
The flow diagram of the selection of studies.

In total, our initial literature search yielded 85 published studies, of which 63 studies were excluded for not investigating the association of GNB3 C825T polymorphism with FD after screening titles and abstracts. After the subsequent reviewing of the remaining 22 articles, 5 were excluded for not being case-control or cohort articles. Seventeen potentially relevant studies were retrieved for full-text evaluation, of which 5 further studies were excluded with insufficient data. Finally, 12 case-control studies with a total of 1109 cases and 2853 controls were included in the present meta-analysis. The study selection is summarized in Figure 1.

The detailed characteristics of all included studies are shown in Table 1. Among the eligible studies, of which eight investigated Asian populations [26, 27, 32, 33, 36–39], three were conducted in American populations [24, 25, 35], and only one study explored a Caucasian population [34]. In addition, eleven studies were published in English, and one was in Chinese [32], representing an international experience from 5 countries. The Rome III criteria were employed to select FD patients among six studies [26, 27, 36–39], while the Rome II criteria were applied in the other six [24, 25, 32–35]. Moreover, the cases from 2 studies suffered from IBS simultaneously [34, 35]. The genotype distribution in the controls of all studies was in agreement with the HWE except for 3 studies [24, 32, 38]. Ten of the included studies were case-controlled in design, and the other two were performed in a cohort way [25, 26].

### 3.2. Overall Analysis

**Figure 2:**
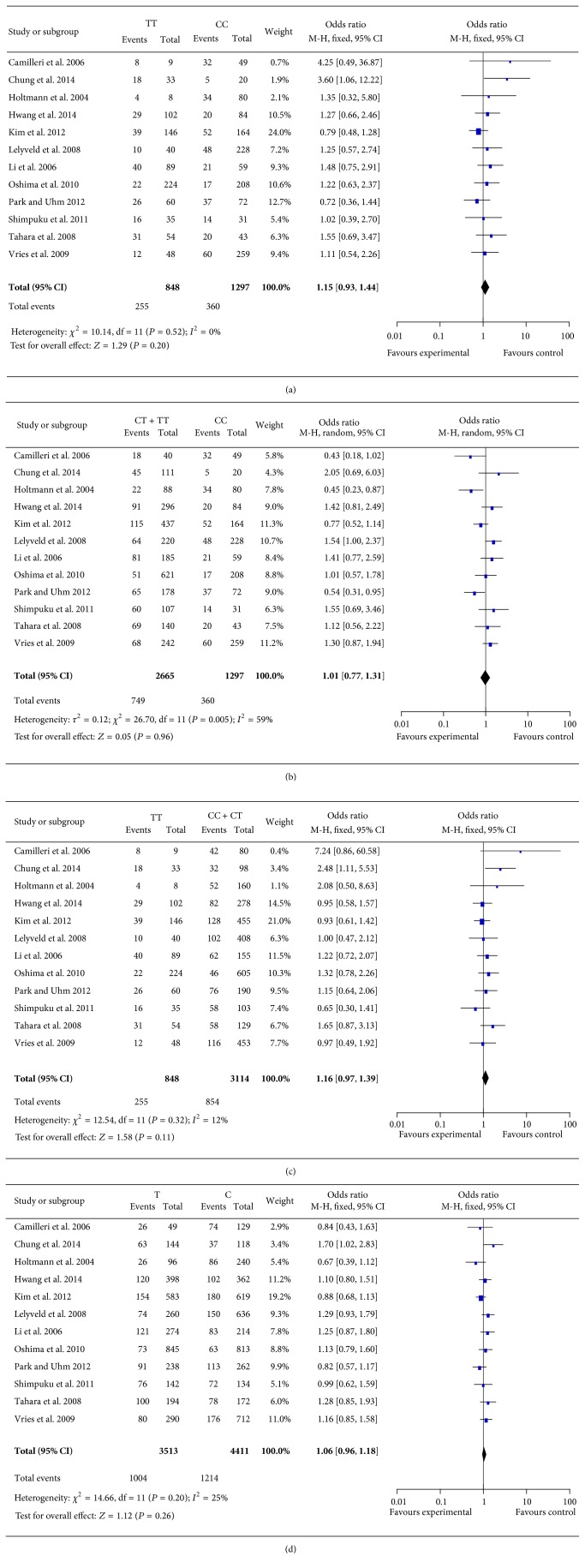
The forest plot for the associations of GNB3 C825T polymorphism and FD risk based on overall analysis. (a) For additive model (CC versus TT). (b) For recessive model (CT + TT versus CC). (c) For dominant model (CC + CT versus TT). (d) For allelic model (C versus T).

Four different genetic models (additive, allelic, dominant, and recessive) were assumed to observe the association between GNB3 C825T polymorphism and FD risk. The main results of the overall analysis are presented in Figure 2 and Table 2. In allelic model (C versus T), the comparison of C with T allele generated a nonsignificant association with FD risk (OR = 1.06, 95% CI: 0.96–1.18, *P* = 0.26). No evidence of significance was identified in additive (CC versus TT, OR = 1.15, 95% CI: 0.93–1.44, and *P* = 0.20) model, as well as in dominant (CC + CT versus TT, OR = 1.16, 95% CI: 0.97–1.39, and *P* = 0.11) model. In one out of the four contrast models considered (recessive model), there was a substantial degree of heterogeneity as indicated by the large value of the *I* ^2^ index (*I* ^2^ = 59%). Thus, the pooled OR was estimated by utilizing the random-effects model under this model. Likewise, in the recessive model (CT + TT versus CC), comparison of CT + TT with CC genotype failed to generate a significant association with FD risk (OR = 1.01, 95% CI: 0.77–1.31, and *P* = 0.96).

### 3.3. Subgroup Analysis

**Figure 3:**
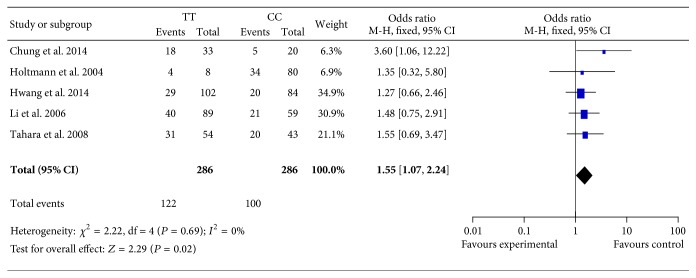
Forest plot of the associations between GNB3 C825T polymorphism and FD risk under additive model based on PCR-RFLP genotyping method.

Considering the fact that the definition of FD, region, and the genotyping method might bias the overall results (Table 2), subgroup analyses were further conducted according to these prespecified covariates, in order to enhance the reliability and stability of this meta-analysis. When stratified by “the definition of FD,” two subtypes of FD were addressed under the utilization of the Rome II and III criteria. However, there was no association among the 4 models of GNB3 C825T polymorphism and FD risk (*P* > 0.05 for all). In the view of region, the subjects who came from Asia were analyzed from the data of 8 studies, failing to yield any significant association between GNB3 C825T polymorphism and FD risk (*P* > 0.05 for all). Likewise, no association was found among the participants from America. The studies were also divided into subgroups based on the genotyping method of the C825T polymorphism. Only two studies were performed in the application of each following genotyping method: direct sequencing, molecular beacon assay, and TaqMan assay, respectively [25–27, 34–36]. Hence, the result of subgroup analysis could not be derived due to the lack of sufficient information among these methods. Consequently, the remaining 5 studies were included for subgroup analysis, using the polymerase chain reaction-restriction fragment-length polymorphism technique (PCR-RFLP) to determine the polymorphism of C825T [24, 32, 33, 38, 39]. With regard to the allelic, recessive, and dominant models, no significant association was observed between GNB3 C825T polymorphism and FD risk (*P* > 0.05 for all). As shown in Figure 3, only the analysis on additive model demonstrated an association between GNB3 C825T polymorphism and significant increasing risk of FD (OR = 1.55, 95% CI: 1.07–2.24; *P* = 0.02).

### 3.4. Sensitivity Analysis

To further strengthen the confidence of current meta-analysis, sensitivity analyses were conducted while restricting the studies on some predefined variables that might hold the potential contribution to the pooled OR of overall results. We excluded studies by Holtmann et al. [24], Li et al. [32], and Chung et al. [38], since the genotype distribution in the control groups deviated slightly from HWE. However, we found that the corresponding pooled ORs were not substantially altered (Table 2). Sensitivity analysis was also conducted by excluding three studies with relatively poor quality (NOS Score < 6), but the results did not change qualitatively by these studies. Similarly, there was little modification of the estimated pooled ORs after exclusion of two studies by de Vries et al. [35] and Lelyveld et al. [34] whose cases suffered from IBS synchronously, indicating that the results of initial analyses were stable.

### 3.5. Publication Bias

Lastly, funnel plots were constructed and Egger's test was employed to assess publication bias of this study. Regarding the overall analysis, only the additive model displayed an asymmetric funnel plot, while Egger's test confirmed the presence of greater publication bias (*P* = 0.015). Additionally, as reflected by Egger's test (Table 2), no statistical evidence of publication bias was revealed regarding the other 3 genotypic models (*P* > 0.05 for all). However, two moderate (*P* = 0.033; *P* = 0.031) and an obvious (*P* = 0.021) publication bias were detected under the additive model among sensitivity analysis based on HWE, overlap disease of IBS, and the NOS Score, respectively.

## 4. Discussion

Functional dyspepsia is a significant chronic disease that leads to reduced quality of life among patients both physically and psychologically. Genetic factors as well as gastrointestinal dysfunction, dysfunction of the autonomic nervous system,* Helicobacter pylori* (*H. pylori*) infection, and serum ghrelin level contribute to the occurrence of FD, and some psychological disturbances together with lifestyle factors may also play a critical role in the etiology of FD.

G-protein regulates the functions of ion channels and protein kinases, comprising a family of ubiquitously distributed signal transduction proteins. Most membrane receptors rely on heterotrimeric G-proteins to activate or inhibit intracellular signaling cascades. Hence, a polymorphism of a G-protein may lead to a wide number of pathophysiological effects, as many hormones, neurotransmitters, and sensory stimuli exert their effects on cells by binding to G-protein coupled receptors. Reportedly, a novel polymorphism of C825T has been detected in exon 10 of GNB3 gene encoding the *β*3 subunit, which was demonstrated to result in a truncated deletion but functionally active splicing variant of 41 amino acids [40]. Cholecystokinin (CCK) is released postprandially from neuroendocrine cells in the duodenal mucosa and delays gastric emptying and promotes sensations of satiety, whose receptor (CCK-1) has been implicated in the generation of dyspeptic symptoms and belongs to the G-protein coupled receptor family [41]. Additionally, there is evidence of CCK hyperresponsiveness in FD patients [42]. Thus, it is conceivable intuitively that, in carriers of the 825T allele, the response via the CCK-1 receptor situated on vagal afferents in the duodenum is enhanced. Moreover, it has been observed recently that T allele carriers of GNB3 C825T are more susceptible to depression and hypertension [43, 44]. Furthermore, homozygous 825T allele carriers correlate with the greatest response to therapy with antidepressive drugs [45] and thiazide diuretics [46], whereas homozygous 825C allele status has shown less responsiveness. On the other hand, individuals with the CT genotype show an intermediate response to these drugs [46]. In this way, the higher prevalence of the 825T allele, which is related to enhanced signal transduction upon G-protein coupled receptor, may be involved in the potential pathophysiological mechanism underlying FD.

Evidence of the correlation between GNB3 C825T polymorphism and FD has been accumulating steadily over the past decade with inconsistent results. Holtmann et al. [24] show that the 825C allele is associated with susceptibility to FD in an American population, whereas three Asian studies together with one Caucasian study show that the 825T allele is a risk factor of FD [26, 33, 34, 38]. Furthermore, two studies recruiting Japanese and Korean participants support such associations of 825CC genotype with the risk of FD [36, 37]. Likewise, both 825C allele and 825T allele are suggested to be associated with FD risk in one study involving American populations [25]. However, the positive findings in above studies failed to be replicated by three other subsequent or simultaneous studies [27, 32, 39]. These contrasting observations may be explained by differences in genotypic composition of populations in different countries, which comprise different racial groups. Overall, via a comprehensive and quantitative meta-analysis with 12 study populations totaling 1109 cases and 2853 controls, we were unable to find significant associations of GNB3 C825T polymorphism with FD risk, and significant between-study heterogeneity in the pooled analyses was found in recessive model (*I* ^2^ = 59%).

Region or ethnicity is generally one of the causes of discrepancies existing in the overall analyses. When it comes to GNB3 C825T polymorphism, the T allele frequency is reported to be higher in Asian populations (42%–53%) than Caucasian populations (27–42%) [47, 48]. Hence, we conducted a subgroup analysis by ethnicity. However, no substantial change was observed, which removed the influence of geography location differentia on the negative results of initial overall analyses. Similarly, the results of subsequent subgroup analysis do not appear to support the notion that the disparity is due to differences of the definition of FD. Genotyping method for gene frequency might be another explanation for the conflicting results; nevertheless, the subgroup analysis focusing on the PCR-RFLP technique indicated no apparent relevance of GNB3 C825T polymorphism to risk of FD in the case of allelic, recessive, and dominant models. But interestingly, a marginally significant association of 825TT genotype with increased 55% FD risk was yielded only under the additive model (OR = 1.55, *P* = 0.02). However, it could not be ignored that the deficiency of studies is embodied in the subgroup analysis limited on genotyping method under the additive model. Besides, 4 out of 5 studies included in the subgroup analysis above are hailed from Asia. Thus, in view of the relatively small sample sizes of this subgroup analysis, the positive analytic results should be interpreted preliminarily and cautiously.

The negative association of all overall analyses discovered here, combined with the nearly analogous outcome of each subgroup analysis, is an obvious indication of the need to perform additional sensitivity analyses, for the sake of enhancing the reliability of present meta-analysis. Statistically significant violations of HWE or magnitude of deviations from HWE may contribute to the problem of replicating postulated gene-disease associations across different studies. Additionally, Trikalinos et al. suggested that gene-disease association meta-analysis should routinely scrutinize the potential impact of HWE violations as well as deviations from the exact frequencies expected under HWE [49]. Accordingly, we restricted the analysis to studies whose controls were in HWE, with little modification of the estimated pooled ORs. Additional cumulative sensitivity analyses were conducted by excluding two studies whose patients were in the condition of concomitant symptoms of FD and IBS and deleting three studies with low quality, respectively. Likewise, persistent and robust results were obtained, demonstrating the precision and stability of the initial overall estimates.

The strength of our study is based on its compliance with criteria for rigorously performing a meta-analysis. Of note, meta-analysis has been regarded as a powerful tool for pooling data from several studies, overcoming the problem of small sample numbers as well as insufficient statistical power of genetic association studies of complex diseases [50]. A previous meta-analysis of studies by Dai et al. [51] revealed that the GNB3 C825T polymorphism was not associated with FD risk except the significant association under an additive model (OR = 0.59, *P* = 0.018), which was completely opposite to our study. As mentioned above and previous studies, the GNB3 825T allele is associated with enhanced G-protein activation and thereby altered signal transduction response, resulting in motor or sensory abnormalities of the gastrointestinal tract. Therefore, it is more reasonable and precise to regard the 825T allele as the susceptible risk gene for FD. But conversely, the 825C allele was employed as the experimental group to evaluate the association between GNB3 C825T polymorphism and the risk of FD in the study by Dai et al., which was unreasonable and may lead to a contrasting observation of the significant OR for the CC genotype. Besides, the sample size (cases: 718; controls: 1988) is too limited to reach conclusive findings regarding this antecedent meta-analysis. Nonetheless, the results of the present meta-analysis should be interpreted within the context of several potential limitations. First of all, although we have made every effort to find suitable studies, we cannot be sure if some appropriate studies were overlooked and there may be other eligible studies that were not published and indexed by electronic databases. Furthermore, meta-analysis can be prone to produce publication bias; despite the literature research of focusing on papers published without language and country restrictions, a palpable publication bias was detected with Egger's test among overall analysis under the additive genetic model (*P* = 0.015). Second, although more participants are employed compared to Dai's study, our analysis is still limited by the relatively small number of included studies. The rarity of included studies would further reduce the importance of the quantitative combination of ORs, while preventing us from constructing more extensive subgroup analysis or sensitivity analysis. Third, the diagnostic criteria for FD are not uniform, with earlier publications adopting Rome II criteria and later ones adopting Rome III criteria (Table 1), inevitably causing selection bias. Finally, there is inadequate and unclear evidence specifically and definitely suggesting which model is propitious to uncover the genetic association of GNB3 C825T polymorphism with FD.

In conclusion, the available evidence of our meta-analysis shows that there are no associations between GNB3 C825T polymorphism and FD risk. Considering the limitations mentioned above, well-designed studies with larger sample sizes are highly desired to validate our findings and elucidate the potential mechanism linking GNB3 to FD.

## Figures and Tables

**Table 1:** Characteristics of included studies in the meta-analysis.

Author	Year	Country	Ethnicity	Mean age (FD/control)	Gender (male/female)	Definition of FD	Genotyping method	Functional dyspepsia				Controls				HWE	NOS Score
								Total	CC	CT	TT	Total	CC	CT	TT		
Holtmann et al. [24]	2004	America	American	46.4 ± 1.9/44.4 ± 1.3	60/108	Rome II	PCR-RFLP	56	34	18	4	112	46	62	4	0	**5**
Camilleri et al. [25]	2006	America	Caucasian	55/60	44/45	Rome II	Direct sequencing	50	32	10	8	39	17	21	1	0.07	**6**
Li et al. [32]	2006	China	Chinese	48.9 ± 9.9/46.2 ± 8.9	112/132	Rome II	PCR-RFLP	102	21	41	40	142	38	55	49	0.01	**5**
Tahara et al. [33]	2008	Japan	Japanese	60.1 ± 13.1/61.1 ± 13.1	130/53	Rome II	PCR-RFLP	89	20	38	31	94	23	48	23	0.84	**7**
van Lelyveld et al. [34]	2008	Netherlands	Caucasian	42.3 ± 1/41.9 ± 1	115/333	Rome II	Molecular beacon assay	112	48	54	10	336	180	126	30	0.25	**6**
de Vries et al. [35]	2009	America	American	49.7 ± 12.3/40.1 ± 12.3	182/319	Rome II	Molecular beacon assay	128	60	56	12	373	199	138	36	0.1	**5**
Oshima et al. [26]	2010	Japan	Japanese	43/45	350/479	Rome III	TaqMan Assay	68	17	29	22	761	191	368	202	0.37	**6**
Shimpuku et al. [36]	2011	Japan	Japanese	59.2 ± 14.2/37.2 ± 9.13	93/45	Rome III	Direct sequencing	74	14	44	16	64	17	28	19	0.32	**8**
Kim et al. [27]	2012	Korea	Korean	49 ± 15/47 ± 15	229/372	Rome III	TaqMan Assay	167	52	76	39	434	112	215	107	0.85	**8**
Park and Uhm [37]	2012	Korea	Korean	11.2 ± 3.6/10.8 ± 3.9	113/137	Rome III	Single base primer extension assay	102	37	39	26	148	35	79	34	0.41	**8**
Chung et al. [38]	2014	Korea	Korean	46.8 ± 15.7/50.5 ± 11.1	62/69	Rome III	PCR-RFLP	50	5	27	18	81	15	51	15	0.02	**8**
Hwang et al. [39]	2014	Korea	Korean	50/54	147/234	Rome III	PCR-RFLP	111	20	62	29	269	64	132	73	0.77	**6**

HWE, Hardy-Weinberg equilibrium; NOS, Newcastle-Ottawa Scale.

**Table 2:** Meta-analysis of the association between GNB3 gene C825T polymorphism and functional dyspepsia risk.

Comparisons	OR (95% CI)	*P* value	*I* ^2^	Egger's test
CC versus TT				
Overall	1.15 (0.93–1.44)	0.20	0	**0.015**
Asia	1.12 (0.87–1.43)	0.37	18%	**0.049**
America	1.34 (0.74–2.42)	0.34	0	0.291
Rome II	1.40 (0.98–1.98)	0.06	0	0.147
Rome III	1.02 (0.77–1.35)	0.87	27%	0.101
PCR-RFLP	1.55 (1.07–2.24)	**0.02**	0	0.320
HWE	1.06 (0.84–1.35)	0.63	0	**0.033**
IBS	1.16 (0.92–1.46)	0.21	1%	**0.031**
NOS Score	1.12 (0.87–1.43)	0.38	15%	**0.015**
CC + CT versus TT				
Overall	1.16 (0.97–1.39)	0.11	12%	0.057
Asia	1.14 (0.94–1.39)	0.19	19%	0.304
America	1.40 (0.80–2.44)	0.24	46%	0.097
Rome II	1.32 (0.98–1.77)	0.07	0	0.126
Rome III	1.08 (0.86–1.35)	0.53	29%	0.475
PCR-RFLP	1.33 (1.00–1.77)	0.05	19%	0.171
HWE	1.08 (0.88–1.32)	0.44	0	0.180
IBS	1.17 (0.97–1.41)	0.10	19%	0.052
NOS Score	1.15 (0.94–1.41)	0.17	31%	0.103
CT + TT versus CC				
Overall	1.01 (0.77–1.31)	0.96	59%	0.818
Asia	1.00 (0.82–1.23)	0.97	42%	0.078
America	0.67 (0.30–1.50)	0.33	80%	0.215
Rome II	0.99 (0.66–1.49)	0.96	67%	0.098
Rome III	1.00 (0.70–1.43)	0.99	52%	0.157
PCR-RFLP	1.11 (0.69–1.80)	0.67	59%	0.790
HWE	1.02 (0.77–1.34)	0.91	56%	0.603
IBS	0.98 (0.73–1.31)	0.87	60%	0.520
NOS Score	1.02 (0.75–1.38)	0.92	56%	0.807
C versus T				
Overall	1.06 (0.96–1.18)	0.26	25%	0.915
Asia	1.06 (0.93–1.20)	0.39	26%	0.082
America	0.97 (0.76–1.25)	0.84	42%	0.384
Rome II	1.14 (0.97–1.33)	0.11	17%	0.094
Rome III	1.01 (0.87–1.16)	0.93	31%	0.190
PCR-RFLP	1.16 (0.97–1.38)	0.10	43%	0.884
HWE	1.04 (0.93–1.17)	0.47	0	0.934
IBS	1.05 (0.94–1.17)	0.40	30%	0.730
NOS Score	1.06 (0.94–1.19)	0.37	24%	0.435

HWE, Hardy-Weinberg equilibrium; IBS, irritable bowel syndrome; PCR-RFLP, polymerase chain reaction-restriction fragment-length polymorphism; NOS, Newcastle-Ottawa Scale.
